# Draft Genome Sequence of *Rhizobium mesoamericanum* STM3625, a Nitrogen-Fixing Symbiont of *Mimosa pudica* Isolated in French Guiana (South America)

**DOI:** 10.1128/genomeA.00066-12

**Published:** 2013-01-24

**Authors:** Lionel Moulin, Damien Mornico, Rémy Melkonian, Agnieszka Klonowska

**Affiliations:** IRD, Laboratoire des Symbioses Tropicales et Méditerranéennes, Campus de Baillarguet, Montpellier, France

## Abstract

*Rhizobium mesoamericanum* STM3625 is a *Mimosa pudica* symbiont isolated in French Guiana. This strain serves as a model bacterium for comparison of adaptation to mutualism (symbiotic traits, bacterial genetic programs for plant infection) between alpha and beta rhizobial symbionts of *Mimosa pudica*.

## GENOME ANNOUNCEMENT

Rhizobia are nitrogen-fixing legume symbionts belonging to the alpha and beta subclasses of *Proteobacteria*, and the names alpha and beta rhizobia are used to distinguish them from each other (1, 2). Alpha rhizobia are common symbionts of most legume species, and the beta rhizobia described so far have an affinity toward the *Mimosa* genus (1). *Mimosa pudica* is a legume species that has the unusual property of interacting with both alpha and beta rhizobia, though diversity studies have shown that alpha rhizobia are less frequent than beta rhizobia on this species (3, 4, 5, 6, 7, 8, 9) and are less competitive for nodulation (10). This plant species thus represents an interesting host for comparative analyses of symbiotic traits and plant-infection genetic programs in the two categories of symbionts.

*Rhizobium mesoamericanum* STM3625 is an alpha rhizobial strain symbiotic of *Mimosa pudica*. The genome of this strain was sequenced to serve as a model bacterium for comparative analyses with beta rhizobia. The STM3625 strain was originally trapped by *Mimosa pudica* from a soil sample in a coastal garden on the east of Cayenne, French Guiana, GPS coordinates 4°56′46′′N, 52°18′03′′W (8). The strain STM3625 is able to efficiently nodulate *M. pudica* and bean, and was included in the *R. mesoamericanum* species (11) by analyses of two housekeeping genes (identity with 16S rRNA and *recA* genes of CCGE501^T^ of 99% and 98%, respectively).

The sequencing of the *R. mesoamericanum* STM3625 genome was obtained using Roche 454 technology (200-bp sequence length) on a genomic DNA mate-pair library of 8 kb, with a 31× final coverage. The assembly was done using Newbler 2.3 (12), leading to 7 scaffolds. A comparison with the genome of *Rhizobium leguminosarum* bv. viciae 3841 led to the identification of one chromosome (RHI3625) and six plasmids (RHI3625p1 to -p6), for a sum of 6,461,927 bp. Each scaffold still contained small gaps (85 holes of mean size 0.1 to 1 kb). Some holes were filled by designed PCR primers on contig ends, PCR, and sequencing by the Sanger method. Finally, the number of gaps (due to mate-pair assembly) corresponded to 34, 13, 20, 7, 9, 2, and 0 holes for RHI3625 and RHI3625p1 to -p6, respectively (a total of 7,872 undetermined bases). Automatic genome annotation was performed using the MaGe annotation server (13) (http://www.genoscope.cns.fr/agc/microscope/mage/) followed by manual annotation. Additional corrections of coding-sequence predictions were performed by using RNAseq data and performed in a separate project. The STM3625 chromosome size is 4,132,041 bases, and plasmids are (from p1 to p6) 1,561,715, 551,965, 108,298, 87,722, 8,157, and 12,029 bp, respectively. The genome has a *G+C* content average of 58.20%. The STM3625 genome contains 1 rRNA operon, 45 tRNA genes, and 6,511 protein-coding sequences (CDSs). The symbiotic plasmid (RHI3625p2) contains two nodulation gene operons and three *nodD* regulators. Two copies of *nodA* were found, one copy in the *nodA1BCSUIJHPQ* operon and another copy close to fatty acid modification genes. The pSym also carries the whole *nif* and *fix* operons for symbiotic nitrogen fixation, and the symbiotic sigma factor *rpoN*.

### Nucleotide sequence accession numbers.

The genome sequence (93 contigs) has been deposited at EMBL/GenBank under accession numbers CANI01000001 to CANI01000092, Bioproject PRJEB128.
